# Schaftoside Interacts With NlCDK1 Protein: A Mechanism of Rice Resistance to Brown Planthopper, *Nilaparvata lugens*

**DOI:** 10.3389/fpls.2018.00710

**Published:** 2018-05-29

**Authors:** Pei-Ying Hao, Ya-Lin Feng, Yi-Shen Zhou, Xin-Mi Song, Hong-Liang Li, Yan Ma, Cheng-Long Ye, Xiao-Ping Yu

**Affiliations:** Zhejiang Provincial Key Laboratory of Biometrology and Inspection and Quarantine, College of Life Sciences, China Jiliang University, Hangzhou, China

**Keywords:** rice, varietal resistance, flavonoids, schaftoside, brown planthopper, CDK1 protein, interaction mechanism

## Abstract

Brown planthopper (BPH) *Nilaparvata lugens* Stål is a serious insect pest of rice in Asian countries. Active compounds have close relationship with rice resistance against BPH. In this study, HPLC, MS/MS, and NMR techniques were used to identify active compounds in total flavonoids of rice. As a result, a BPH resistance-associated compound, Peak 1 in HPLC chromatogram of rice flavonoids, was isolated and identified as schaftoside. Feeding experiment with artificial diet indicated that schaftoside played its role in a dose dependent manner, under the concentration of 0.10 and 0.15 mg mL^-1^, schaftoside showed a significant inhibitory effect on BPH survival (*p* < 0.05), in comparison with the control. The fluorescent spectra showed that schaftoside has a strong ability to bind with NlCDK1, a CDK1 kinase of BPH. The apparent association constant K_A_ for NlCDK1 binding with schaftoside is 6.436 × 10^3^ L/mol. Docking model suggested that binding of schaftoside might affect the activation of NlCDK1 as a protein kinase, mainly through interacting with amino acid residues Glu12, Thr14 and Val17 in the ATP binding element GXGXXGXV (Gly11 to Val18). Western blot using anti-phospho-CDK1 (pThr14) antibody confirmed that schaftoside treatment suppressed the phosphorylation on Thr-14 site of NlCDK1, thus inhibited its activation as a kinase. Therefore, this study revealed the schaftoside-NlCDK1 interaction mode, and unraveled a novel mechanism of rice resistance against BPH.

## Introduction

Brown planthopper *Nilaparvata lugens* Stål is one of the most serious insect pests of rice (*Oryza sativa* L.) in Asian countries. Outbreaks of BPH can cause severe yield losses of rice and have occurred quite frequently in recent years (Bottrell and Schoenly, 2012; Hu et al., 2013). Breeding resistant rice varieties is an important strategy to control BPH, but the resistance of rice is often broken down rapidly owning to the virulence variation of this insect (Park et al., 2007; Liu et al., 2015). The interaction mechanism between rice and BPH still remained unclear, which hampers the sustainable utilization of rice resistance. Currently, controlling of BPH largely relies on extensive application of chemical insecticides, and a promising approach to search for novel insecticides is based on secondary metabolites produced by plants. Therefore, it is critical to identify some active compounds in resistant rice and explore the mechanism of interaction between the active compound and the BPH.

Plants in natural and agricultural ecosystems are constantly exposed to diverse attackers, such as herbivores, pathogens and viruses (Erb et al., 2011; Kamphuis et al., 2013). The secondary metabolites including phenolics, alkaloids, peptides and essential oils produced by plants have long been regarded important for plant defense against herbivores (Farooq et al., 2011; Sun et al., 2013). Flavonoids are a group of plant polyphenolic secondary metabolites, and these compounds consist of two benzene rings connected by a three carbon chain, forming a chemical structure with three rings (C6-C3-C6). The flavonoids can be divided into six major subtypes, which include flavones, chalcones, isoflavonoids, flavonones, anthocyanins, and anthoxanthins (Petrussa et al., 2013). Flavonoids play important roles in plant adaptation to external environments, including interaction with pests (Simmonds, 2001; Nenaah, 2013). For example, isorhamnetin-3-sophoroside-7-glucoside and kaempferol-3,7-diglucoside have been reported as feeding deterrents against *Mamestra configurata* (Onyilagha et al., 2004), rutin and quercetin were found toxic to *Spodoptera litura* larvae (Su et al., 2018), vitexin and vitexin-2-O-arabinofuranoside in *Basella alba* leaves impaired the growth of *Spodoptera litura* larvae (Aboshi et al., 2018).

Rice plants are rich in flavonoids, and some flavonoids are implicated in rice resistance to BPH. The flavonoids, tricin, can defend the rice plant against infestation by the BPH (Zhang et al., 2017). Some flavonoid glycoside compounds, such as apigenin-C-glycosides, can affect the feeding behavior of the BPH and result in death of the insect (Grayer et al., 1994; Stevenson et al., 1996; Simmonds, 2001). In our previous study, the same amount of total flavonoids extracted from different rice varieties showed different effects on the survival of BPH, indicating that some key compounds were present in total flavonoids responsible for the resistance of rice to BPH (Zhou, 2011). Since it has been estimated that the flavonoids family possess more than 10,000 members in plant kingdom (Seo et al., 2011), it is necessary to further study the flavonoids in different rice varieties and identify some active compounds. It’s also important to explore the interaction property between the identified active compound in flavonoids and the target in BPH for understanding the resistant mechanism of rice.

Cyclin-dependent kinase 1 (CDK1), also called p34^cdc2^, plays a vital role in regulating the cell cycle as a serine/threonine kinase (Morgan, 1995). Besides, CDK1 is also involved in diverse physiological processes. For example, Cdk1 play roles in cell adaptive response to stress (Candas et al., 2013), regulation of mitochondrial preprotein translocase (Harbauer et al., 2014), and enhancing mitochondrial bioenergetics (Qin et al., 2015). Knockdown of CDK1 caused cell death in *Drosophila melanogaster* (Björklund et al., 2006). Some flavonoids, especifically chalcones and flavones containing nitrogen, have been reported as CDK1 inhibitors (Navarro-Retamal and Caballero, 2016). Therefore, CDK1 in BPH (NlCDK1) might be a potential target of rice flavonoids, and worthy being further studied.

Although flavonoids are abundant in rice plants, it is still uncertain whether the composition and content of flavonoids in different rice varieties are same or not, especially, the binding property of resistance-related flavonoids with NlCDK1 remains unclear. In the present work, we analyzed the flavonoids from different rice varieties by comparing the HPLC profiles, isolated and identified schaftoside as an active compound associated with BPH-resistance. We also explored the molecular mechanisms involved in the interaction between schaftoside and NlCDK1. Therefore, this research not only identified a promising compound schaftoside which might be developed as new insecticide to control BPH, but also clarified the interaction between schaftoside and NlCDK1, thus untraveled a novel mechanism of rice resistance against BPH.

## Materials and Methods

### Plants and Insects

Colonies of BPH were maintained on Taichung Native 1 (TN1, a variety without resistant gene to BPH) rice seedlings. Rice varieties of TN1 and Xiushui 11 (without resistance gene to BPH) are susceptible, while Mudgo (with *Bph 1*), ASD7 (with *bph 2*) and Rathu Heenati (RHT, with *Bph 3*) exhibit resistance to BPH of TN1 colony in the seedling bulk test. Unless otherwise stated, rice plants used in experiments were three-leaf to four-leaf stage, and test BPH were 2-3 instar nymphs. Rice plants and BPH were maintained in a phytotron at 26 ± 2°C and 75–85% RH with a 14:10 h L:D photoperiod.

### Total Flavonoids Extraction and Quantification

Total flavonoids were extracted from rice plants according to previous methods (Pereira et al., 2012) with some modifications, as following: non-infested leave sheathes of rice were sampled at the tillering stage about 2 months after transplanting, blanched at 105°C for 30 min and dried at 60°C to constant weight. The dried plant material was powdered and extracted ultrasonically under an optimized condition: 70% ethanol solution, a solid-to-liquid ratio of 1:25 (m/v), a power of 250 w, and an extracting time of 50 min (Zhou et al., 2011). The collected residue was extracted in the same condition for two more times. The filtrates were combined and defatted with *n*-hexane (1:1, v:v) for two times. The filtrates were concentrated by vacuum evaporation in rotavapor and adjusted the volume of total flavonoids to 10 mL. The prepared total flavonoids were filtered through a nylon membrane filter (0.45 μm pore size). The content of total flavonoids extracts was examined using the sodium nitrite-aluminum nitrate colorimetric method using rutin as a reference substance. A calibration curve of rutin was prepared as described by Zhou et al. (2011). Five biological replicates were analyzed for each variety, and each biological replicate includes at least 10 rice plants.

### HPLC Chromatographic Condition and Quantification of the Target Compound

High performance liquid chromatography analysis was carried out on a VARIAN ProStar 240 instrument equipped with a binary pump, a PDA Varian Prostar detector. The conditions were modified as previously described (Gouveia and Castilho, 2010; Pereira et al., 2012). The wavelength range was set at 220–400 nm, monitored at 350 nm. Separation was performed using a Hypersil Gold column (150 mm × 4.6 mm, 5 μm), with a sample injection volume of 10 μL. CH3CN (A) and water containing 0.1% formic acid (B) were used as chromatographic eluent, and the program for eluenting gradient was set as follows: 8–35% A (0–60 min), 95% A (61–70 min), 8% A (71–80 min). The flow rate of mobile phase was 1.0 mL min^-1^, and the chromatogram was recorded at 350 nm. Column temperature was controlled at 30°C.

The target compound (Peak 1) in rice total flavonoids related to BPH-resistance was selected by comparing the HPLC profiles of different rice varieties. For relative quantification of the target compound Peak 1 in rice total flavonoids, a calibration curve was obtained by diluting an original total flavonoids extract of RHT rice variety (relative concentration, 1.0) into a series solution with different concentrations (0.5, 0.25, 0.125, and 0.0625), and the calibration curve was constructed by plotting the peak-areas to the relative concentrations of the target compound Peak 1. The concentration of Peak 1 in the original total flavonoids selected is high enough, to ensure that the relative concentration of Peak 1 in all test samples will not exceed 1.0. Relative quantification for Peak 1 of different rice varieties was achieved by using the calibration curve. Five biological replicates were analyzed for each variety.

### LC-ESI-MS/MS Analysis

LC-ESI-MS/MS analysis was carried out based on the method modified from Simirgiotis et al. (2013) as follows: a mass spectrometer of Esquire 4000 Ion Trap (Bruker Daltonics, Bremen, Germany) was connected to an Agilent 1100 HPLC instrument (Agilent Technologies, Waldbronn, Germany) with ESI interface. Data acquisition of full scan was performed from m/z 50 to m/z 2000 in a negative ion mode. Nitrogen was used as nebulizer gas with electrospray needle 4500 V. Using ultrahigh pure helium as the collision gas, induced dissociation spectra were obtained with fragmentation amplitude of 1.00 V (MS/MS). HPLC analysis was carried out using the condition as above of HPLC chromatograph.

### Purification of the Active Compound From Flavonoids

To obtain some pure sample for NMR analysis, the target compound of peak 1 was purified from total flavonoids extract in the following way: total flavonoids extract was firstly separated with Sephadex LH-20 medium pressure chromatography (1000 mm × 30 mm ID), the eluent is methanol/water (1/9-7/3) with a flow rate of 2.0 mL min^-1^, loading: 1.0 g. Sample was collected in the same conditions for 3 times. Crude extract of peak 1 in HPLC chromatogram was refined with Medium Pressure Chromatography (400 mm × 10 mm ID, Merck C18, 40–60 μm), eluent is acetonitrile (CH3CN)/water 1:9, and the flow rate is 1.0 mL min^-1^. By evaporating the solvent, the collected fraction was condensed to approximately 1 mL. The supernatant was removed and the precipitated material was solubilized in DMSO-d_6_ for following NMR analysis (Pereira et al., 2012; Simirgiotis et al., 2013).

### Structural Identification by NMR

^1^H and ^13^C NMR spectra of the purified peak 1 compound were recorded on a Bruker Avance 500 spectrometer at 500 and 125 MHz, respectively. DMSO-d_6_ was used as solvent. Chemical shift references were reported from the solvent resonances of DMSO-d_6_ at δ_H_ 2.50 and δ_C_ 39.5, relative to the internal standard tetramethylsilane TMS (Pereira et al., 2012).

### Effect of Schaftoside on BPH Survival

Schaftoside (purity >98%, Chengdu Biopurify Phytochemicals Ltd., Chengdu, China) was added into the artificial diet D-97 (Fu et al., 2001) to examine its effect on BPH. The final concentrations of schaftoside were adjusted to 0.05, 0.1, and 0.15 mg/mL for different treatments. Feeding sack was made using two layers of stretched Parafilm^®^ M film, and 20 μL artificial diet with schaftoside was sandwiched between the films. A glass chamber (2.5 cm × 15 cm), each containing 10 nymphs of 2nd instar, was covered with feeding sacks at both ends. At the same time, D-97 artificial diet without schaftoside was used as the control. The artificial diet with or without schaftoside was renewed every day to keep fresh. Insects were maintained at a temperature of 26 ± 2°C, 70% RH, and a 16:8 L:D photoperiod. Survival rates were recorded every 5 days. Ten biological replicates were analyzed for each treatment.

### Preparation of Recombinant NlCDK1 Protein

The CDS for recombinant NlCDK1 protein expression were amplified by PCR, according to the *NlCDK1* sequence (GeneBank: KX138392) previously submitted to NCBI databases^1^ by our group. The forward primer was NlCDK1-F (5′-GCGGATCCATGAATTCCTACGACATGCTTGAG-3′) containing the *Bam*H I restriction site (underlined), and the reverse primer was NlCDK1-R (5′-GGAAGCTTTTAATCGTAGATATCAGCACCGGG-3′) containing the *Hin*d III restriction site (underlined). The amplified fragments was digested with the corresponding restriction enzymes (*Bam*H I and *Hin*d III) and subcloned into vector pET32a by T4 DNA ligase overnight (for 12 h) at 16°C. The recombinant plasmid was sequenced and transformed into the *Escherichia coli* strain BL21 (DE3) for expression of recombinant protein. The proteins were induced with 0.5 mM isopropyl β-D-thiogalactopyranoside (IPTG) overnight at 15 °C. The recombinant protein with NlCDK1 was purified by ProteinIso^®^ Ni-NTA Resin (TransGen, Beijing, China), and dialyzed against a phosphate buffer saline (PBS, pH7.4). The protein samples were examined by 12% SDS–PAGE, stained with Coomassie Brilliant Blue G-250. BeyoColor^TM^ Prestained Color Protein Marker P0076 (Beyotime, Shanghai, China) was use as molecular weight markers.

### Fluorescent Spectral Experiment

The fluorescent quenching spectra were used to analyze the interaction between schaftoside and NlCDK1 protein. Fluorescence was detected on a Spectrofluorophotometer RF-5301PC (Shimadzu, Japan), which is equipped with a xenon lamp source. To record the fluorescent quenching spectra, the stock solution of NlCDK1 was diluted into working solution with a concentration of 0.24 × 10^-6^mol L^-1^. Afterward, schaftoside was titrated into each working solution of NlCDK1 in the quartz cell. The mixture of schaftoside and NlCDK1 protein were excited at 281 nm, AND the fluorescent emission spectra were recorded at the wavelength of 290–500 nm.

### Molecular Docking Analysis

For docking calculations, the 3D structure of NlCDK1 protein model was firstly predicted by SWISS-MODEL^2^ online using the crystal structure of CDK1 (PDB entry code, 4y72.1.A) as the template. Docking was performed online using DockingServer^3^.

### Western Blot Analysis

Brown planthopper was treated with schaftoside by feeding the nymphs with artificial diet as described above. Western blot was performed according to the method modified from Hao et al. (2015). At day 10, about 25 nymphs of each biological replicate were homogenized in 1 × PBS, and then added 2 × SDS sample buffer. The lysate protein were boiled for 5 min, and centrifuged at 10,000 × *g* for 5 min. Protein samples of 10 μl were loaded onto the SDS gels, electrophoresis for 90 min, and transferred to polyvinylidene difluoride membranes. TBST (0.1% Tween 20 in TBS) and 5% non-fat powdered dry milk (w/v) were used to block the blots for 2 h at room temperature. Primary antibody anti-phospho-CDK1 (pThr14, Sigma-Aldrich, MO, United States) at a dilution of 1:500 was then incubated with the blot for 12 h at 4°C in a TBST solution to probe pThr14 of NlCDK1. The membrane was washed with TBST for 10 min 3 times, and then incubated in the horseradish peroxidase-linked secondary antibody (Solarbio, Beijing, China) at a dilution of 1:1000. The membrane was washed 3 times, detected using the DAB Horseradish Peroxidase Color Development Kit (Sangon Biotech, Shanghai, China), and imaged with a camera equipped in a mobile phone.

### Statistical Analysis

Content of total flavonoids and Peak1 (schaftoside) in different rice varieties was analyzed using one-way ANOVA and *post hoc* analysis with Tukey’s HSD test. Effect of schaftoside on survival rate of BPH was analyzed by Tukey’s test at each sampling time. All analysis was performed with the SPSS program (SPSS 19.0).

## Results

### Content of Total Flavonoids in Different Rice Varieties

**FIGURE 1:**
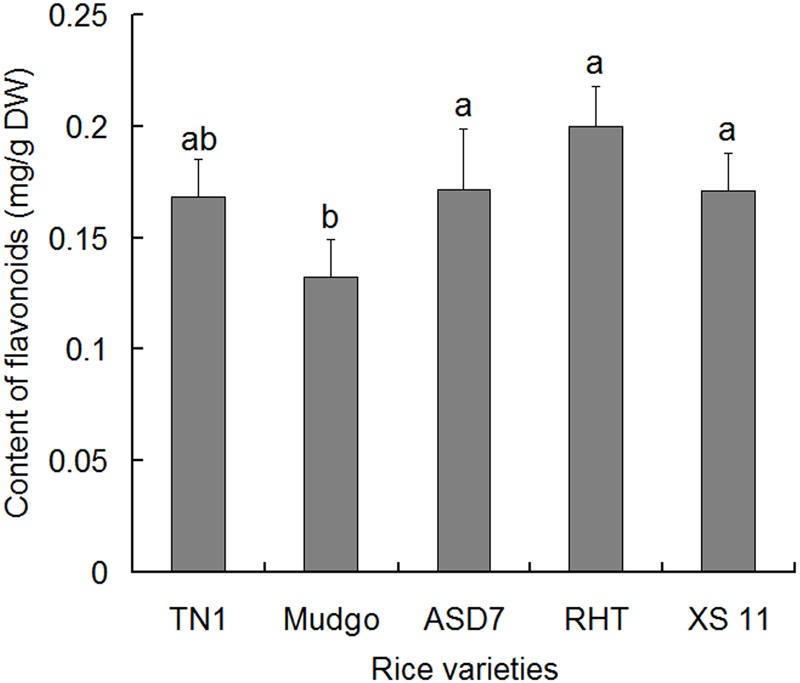
Content of total flavonoids in the leaf sheathes of different rice varieties. Data were presented as means ± SD (*n* = 5). Data were analyzed using one-way ANOVA and post-hoc analysis with Tukey’s HSD test, and relative content with the same letter were not significantly different (*P* < 0.05). DW, dried weight.

Total flavonoids were extracted from rice leaf sheathes and determined using rutin as a reference substance. In general, the content of total flavonoids between the susceptible varieties (TN1 and Xiushui 11) and the resistant varieties (ASD7 and RHT) did not show significant differences, except that the content in resistant variety Mudgo was relatively lower (**Figure 1**). It suggested that there was no significant correlation between the content of total flavonoids and the resistance of rice varieties. Therefore, it is necessary to further explore whether the active compounds in total flavonoids of different varieties are different in content, and contributed different resistance to rice against BPH.

### HPLC Fingerprint Analysis

**FIGURE 2:**
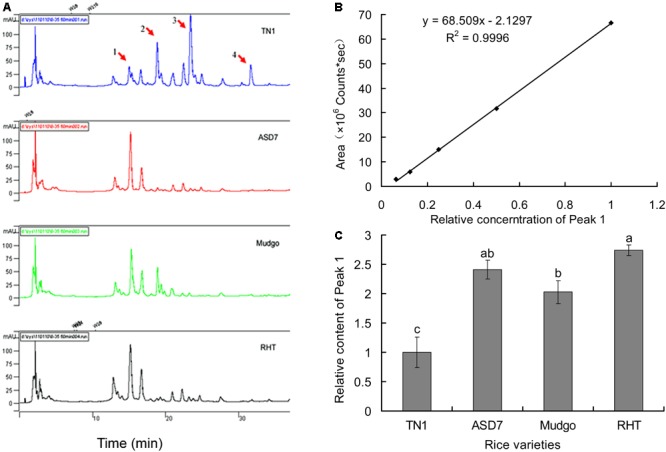
Determination of Peak 1 related compound in rice flavonoids by HPLC. **(A)** HPLC fingerprints of flavonoids extract from different rice varieties. **(B)** A calibration curve constructed by plotting the peak-areas to the relative concentrations of Peak 1 related compound. **(C)** Relative content of Peak 1 related compound in flavonoids extract from different rice varieties, calculated according to the calibration curve in **(B)**. Data was analyzed by ANOVA with Tukey’s HSD tests and relative content with the same letter were not significantly different (*P* < 0.05). Data were presented as means ± SD (*n* = 5).

High performance liquid chromatography fingerprints of total flavonoids from different rice varieties were established to identify the active compound associated with the rice resistance against BPH. In general, four absorption peaks (Peak 1 to 4) in HPLC profiles showed obvious differences between the susceptible TN1 and resistant varieties (ASD7, Mudgo and RHT), whereas only one peak (Peak 1) had a positive correlation with the varietal resistance to BPH (**Figure 2A**). Therefore, Peak 1 related compound was selected as a candidate for further analysis. A calibration curve of Peak 1 was established for HPLC analysis, and the result showed that a good linearity was obtained between the peak area (*y*) and the relative concentration (*x*) of Peak 1 (**Figure 2B**). The relative concentration (*x*) of Peak 1 related compound in different rice varieties was determined according to the corresponding area (*y*) of Peak 1 in the HPLC chromatogram using the equation *y* = 68.509 x - 2.129 (*R*^2^ = 0.9996). It showed that the relative content of Peak 1 was higher in resistant varieties than that in susceptible TN1 (set as 1.0), with a variation ranging from 2.0 (Mudgo) to 2.7 (RHT). It suggested that the relative content of Peak 1 in different rice varieties were generally consistent with the resistance of rice (**Figure 2C**), so the Peak 1 related compound should play a key role in the resistance of rice to BPH.

### HPLC-DAD-ESI-MS-MS Identification of Peak 1 Related Compound

**FIGURE 3:**
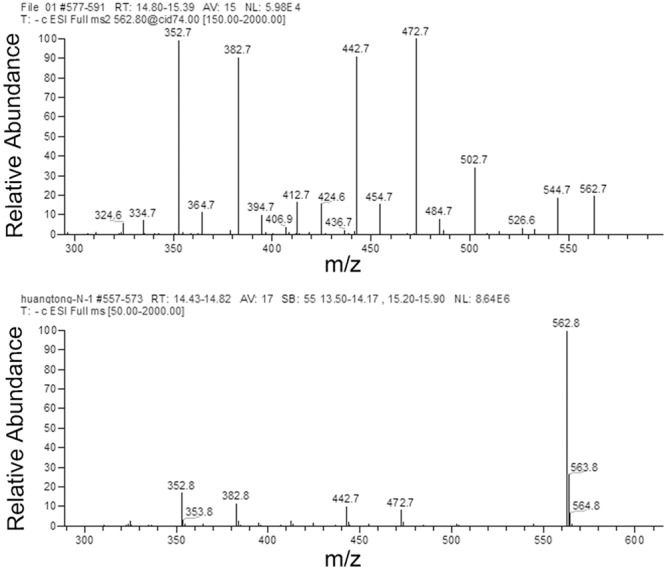
MS/MS of the pseudomolecular ions [M-H]^-^ of Peak 1 related compound.

The flavonoids in rice leaf sheathes were investigated by HPLC-DAD-ESI-MS-MS. For MS analysis, detection of Peak 1 related compound was performed in ESI negative modes. Peak 1 with a [M-H]^-^ ion at m/z 563 produced MS^2^ ions at m/z 545, 503, 473, 443, 383, and 353 (**Figure 3**). According to previous references (Bakhtiar et al., 1994; Ferreres et al., 2003), Peak 1 was concluded to be 6-*C*-arabinosyl-8-*C*-glucosyl apigenin or 6-*C*-glucosyl- 8-*C*-arabinosyl apigenin. However, it was still difficult to identify the exact structure of Peak 1 related compound only by HPLC-ESI-MS-MS analysis. Further elucidation by a more powerful tool, such as NMR spectroscopy was necessary in the following steps to describe an unambiguous structure about Peak 1.

In addition, three other peaks 2, 3, and 4 also showed clear difference between the susceptible rice variety TN1 and resistant varieties, but these peaks didn’t have a positive correlation with the varietal resistance of rice to BPH. Peak 2 with a [M-H]^-^ ion at m/z 533 yielded MS^2^ ions at m/z 443, 473, 383, 353 and 515. Peak 3 yielded an ion at m/z 491 which produced an MS^2^ ion at m/z 329. Peak 4 yielded a [M-H]^-^ ion at m/z 739 and produced MS^2^ ions at m/z 563, 383 and 293 (Supplementary Figures S1–S3).

### Purification and NMR Analysis of Peak 1 Related Compound

**FIGURE 4:**
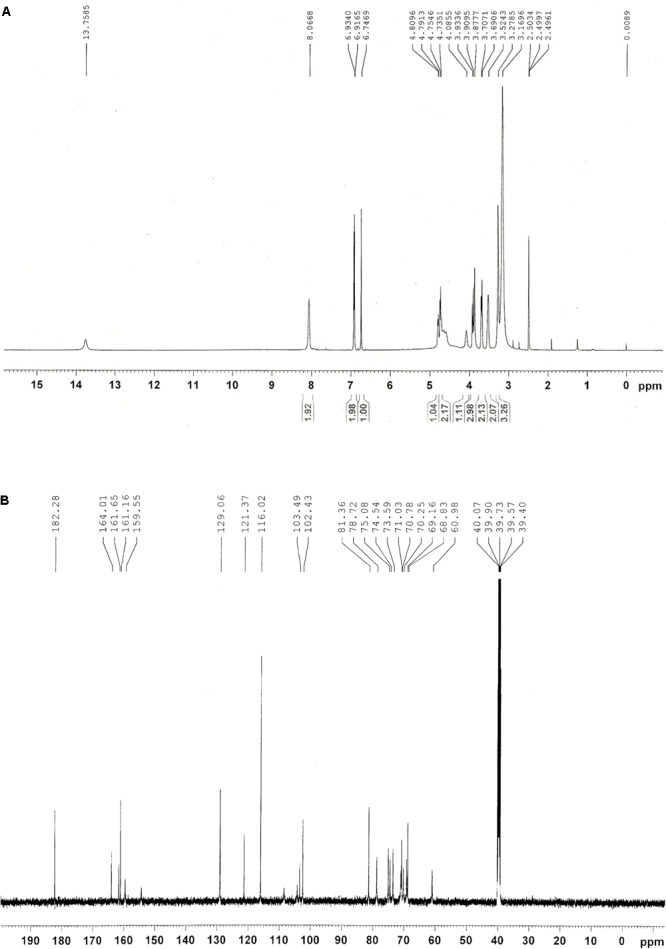
NMR spectra of Peak 1 related compound schaftoside. **(A)** ^1^H NMR spectra; **(B)** ^14^C NMR spectra.

Among four HPLC peaks (Peak 1-4) of total flavonoids extract, only Peak 1 showed a positive correlation with the resistance of rice to BPH (**Figure 2A**), so Peak 1 related compound was separately collected and purified by HPLC. The purified compound of Peak 1 was then elucidated by NMR to identify its exact structure from two candidate compounds 6-*C*-arabinosyl-8-*C*-glucosyl apigenin and 6-*C*-glucosyl-8-*C*-arabinosyl apigenin. According to the NMR spectra of Peak 1 (**Figure 4**), the ^1^H and ^13^C NMR chemical shifts were assigned and showed in **Table 1**. The ^1^H-NMR and ^13^C-NMR data totally agreed with the published data of 6-*C*-beta-*D*-Glucosyl-8-*C*-alpha-L-arabinosylapigenin (schaftoside), previously isolated from several plants and unambiguously elucidated by NMR spectroscopy (Xie et al., 2003; Dou et al., 2007; Simirgiotis et al., 2013).

**Table 1:** Assignments of ^1^H and ^13^C NMR chemical shifts of Peak 1.

Position (H) (500 MHz)	Chemical shift δ, ppm (60°C)	Position (C) (125 MHz)	Chemical shift δ, ppm (60°C)
1H, br s, 5-OH	13.75	C-4	182.1
2H, H-2′,6′	8.07	C-2	163.8
2H, d, *J* = 9.0 Hz, H-3′,5′	6.92	C-7	161.4
1H, s, H-3	6.75	C-5	160.9
1H, d, *J* = 9.2 Hz, H-A1	4.80	C-4′	159.3
1H, d, *J* = 9.8 Hz, H-G1	4.74	C-9	154.1
m, sugar-H	4.09∼3.15	C-2′, 6′	128.8
		C-1′	121.1
		C-3′, 5′	115.8
		C-6	108.2
		C-8	104.1
		C-10	103.3
		C-3	102.2
		G-5	81.1
		G-3	78.5
		A-1	74.9
		G-1	73.4
		A-3	74.3
		G-2	70.8
		A-5	70.6
		G-4	70.0
		A-2	68.9
		A-4	68.6
		G-6	60.8

**FIGURE 5:**
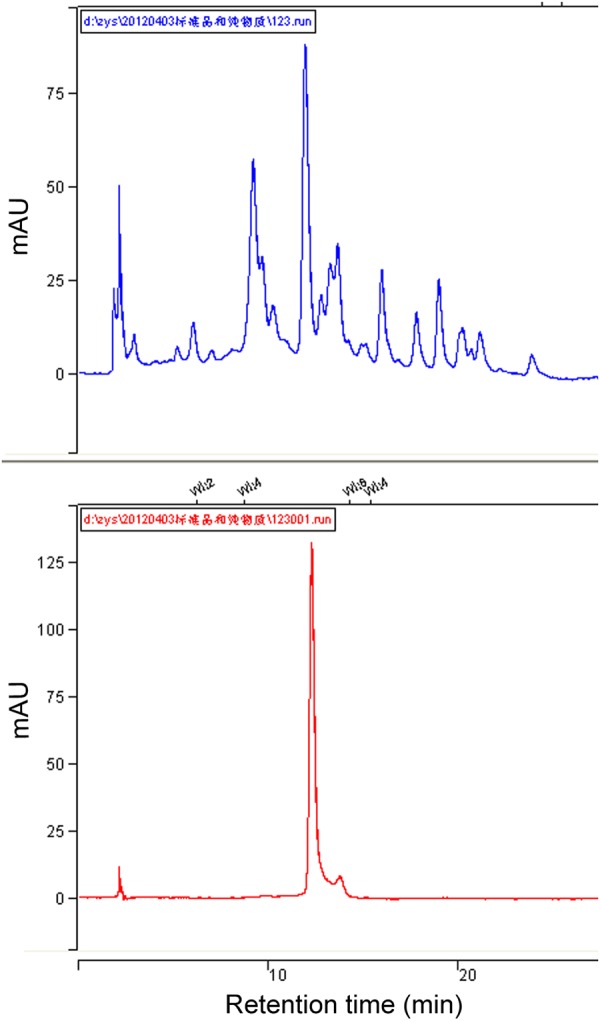
HPLC chromatographs of total flavonoids and the standard sample of schaftoside. Showing the Peak 1 related compound in the flavonoids of RHT rice variety (upper) has the same retention time with the standard sample of schaftoside (lower).

To further confirm that Peak 1 related compound was schaftoside, the standard sample of schaftoside was used as reference to compare with Peak 1 using HPLC technique. The result showed that Peak 1 related compound has same retain time with the standard sample of schaftoside (**Figure 5**). Therefore, Peak 1 related compound was identified as schaftoside.

### Effects of Schaftoside on the Survival Rate of BPH

**FIGURE 6:**
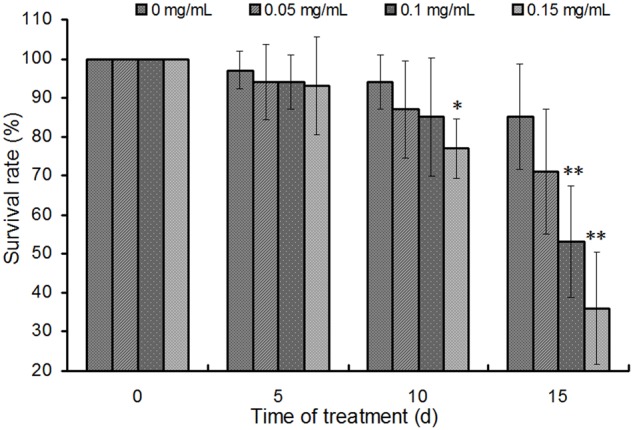
Effects of schaftoside treatment on BPH. The results were expressed as the means ± SD from 10 independent experiments with 10 glass tubes (10 nymphs in each tube) examined for each treatment. The survival rates were analyzed by one-way ANOVA and Tukey HSD at each sampling time, and the asterisks on the bars indicated significant differences between the control (0 mg/mL) and the corresponding treatment group (^∗^*p* < 0.05, ^∗∗^*p* < 0.01).

Schaftoside was dissolved in artificial diet to test its activity on BPH. Generally, survival rates of BPH showed a decline trend in schaftoside treatment groups as well as the control (0 mg mL^-1^) (**Figure 6**). The results also showed that the schaftoside affected BPH survival rate in a dose-dependent way. The treatment with 0.15 mg mL^-1^ schaftoside showed a significant difference at day 10 (*p* < 0.05), while there were no difference between the control and the treatment group of 0.05 mg mL^-1^ or 0.10 mg mL^-1^. At day 15, BPH survival rate in 0.15 mg mL^-1^ schaftoside treatment group showed a large decrease to 36%, while the control still remained at a higher level of 85% (**Figure 6**), suggesting that schaftoside had a strong lethal effect on BPH at this concentration.

### Expression and Purification of NlCDK1

**FIGURE 7:**
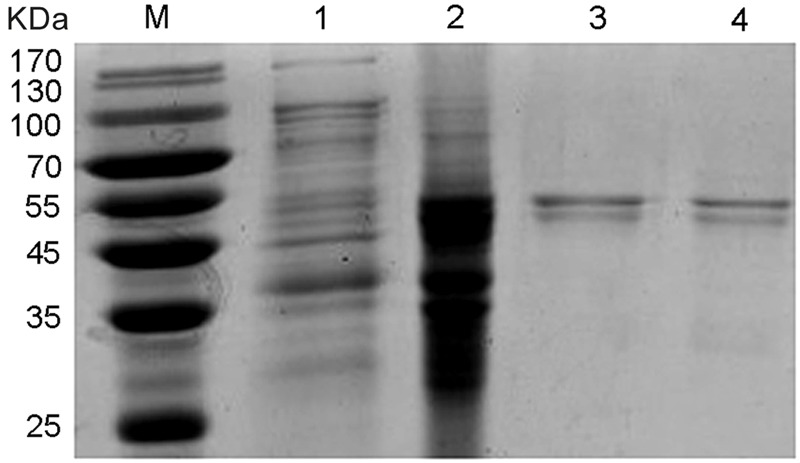
SDS–PAGE gel analysis of recombinant protein NlCDK1. The expression of NlCDK1 expressed in *E. coli* strain BL21(DE3)/pET32a was induced by 0.5 mM IPTG. The samples were analyzed in 12% SDS–PAGE and stained with Coomassie Brilliant Blue G-250. M: molecular weight markers; Lane 1: total cell lysate of BL21(DE3)/pET32a prior to induction; lane 2: protein of total cells after IPTG induction for 12 h; lane 3: purified and denatured NlCDK1 protein in inclusion bodies; lane 4: purified and refolded NlCDK1 protein.

The competent *E. coli* BL21 (DE3) with recombinant plasmids of pET32a /NlCDK1 were induced by IPTG, and the recombinant proteins were then purified from the protein lysate using Ni^2+^-NTA affinity chromatography column. The purified protein, as well as the protein lysates of the crushed bacteria, was analyzed by 12% SDS–PAGE. The result showed that the target recombinant proteins with a molecular weight of about 55 kDa were obtained (**Figure 7**). The recombinant protein contains a fusion protein with histidine marker (about 20.4 kDa) encoded by the pET-32a(+) vector, so the molecular weight of the target NlCDK1 protein is very close to the theoretical value 34.7 kDa, indicating that NlCDK1 was successfully expressed and purified.

### Binding Property of NlCDK1 With Schaftoside

**FIGURE 8:**
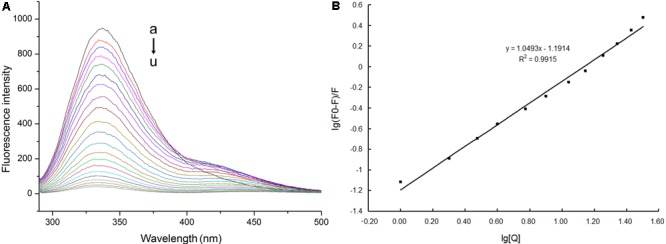
Fluorescence spectra and plots of log[(*F*_0_-*F*)/*F*] versus log[*Q*] for NlCDK1 binding with schaftoside. **(A)** C (schaftoside)/(μmol L^-1^): a, 0; b, 0.5; c, 1.0; d, 2.0;e, 3.0; f, 5.0; g, 7.0; h, 10.0; i, 13.0; j, 17.0; k, 21.0; l, 28.0; m, 35.0; n, 45.0; o, 55.0;p, 67.0; q, 79.0; r, 93.0; s, 107.0; t, 123.0; u, 139.0. **(B)** C (NlCDK1) = 0.24 × 10^-6^ mol L^-1^; pH = 7.4; ex = 281 nm. The increasing concentration of schaftoside **(B)** was in accordance with that in the fluorescence spectra **(A)** when it was titrating from a to l; log[(*F*_0_-*F*)/*F*] = log*K*_A_ + *n*log[*Q*] was presented as *y* = 1.0493*x*–1.1914.

The fluorescent spectra were obtained by titrating schaftoside into the recombinant NlCDK1 protein solution. The fluorescent intensity of NlCDK1 regularly dropped at 335 nm with the titrating of schaftoside, and the emission peaks of the protein showed a very similar pattern at the different concentrations of schaftoside (**Figure 8A**). This reflected that non-fluorescent complexes were generated when schaftoside reached to NlCDK1 protein. Therefore, schaftoside has a strong ability to bind with NlCDK1.

NlCDK1 protein binding with schaftoside was further studied by the equation: log[(*F*_0_-*F*)/*F*] = log*K*_A_ + *n*log[*Q*]. *F*_0_ is the fluorescent intensity without a quencher (Q), while F stands for the fluorescent intensity at [Q] concentration of a quencher added. K_A_ is the apparent association constant, and *n* stands for the number of binding sites per protein. Both K_A_ and *n* were obtained once a plot of log[(*F*_0_ - *F*)/*F*] versus log[*Q*] was constructed (**Figure 8B**). In Figur 8, log[(*F*_0_-*F*)/*F*] = log*K*_A_ + *n*log[*Q*] was presented as *y* = 1.0493*x*-1.1914. According to the double logarithm equation, the apparent association constant K_A_ for NlCDK1 binding with schaftoside is calculated as 6.436 × 10^3^ L/mol, and the number of binding sites per protein *n* is 1.0493, which means that each NlCDK1 protein can bind with at least one schaftoside.

### Molecular Docking

**FIGURE 9:**
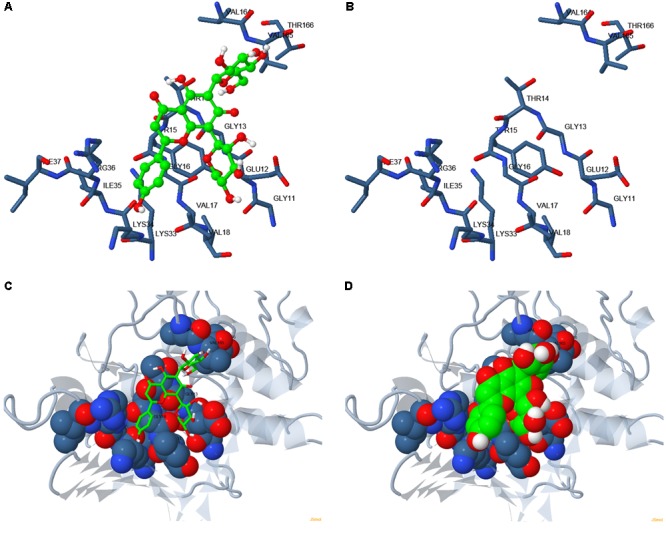
Binding mode of schaftoside to NlCDK1 binding sites. **(A,C,D)** Schaftoside binding with NlCDK1. **(B)** The same amino acid residues located close to the binding regions of NlCDK1 as shown in **(A)**, without binding schaftoside. Carbon atoms in NlCDK1 and schaftoside are shown in blue and green, respectively. Colors of other atoms are as follows: oxygen (red), nitrogen (light blue). In **(C,D)**, gray color represented for non-binding regions of NlCDK1 (local spacial structure).

Based on the structure of CDK1 protein (PDB entry code: 4y72.1.A) available in the Protein Data Bank archive^4^, the tertiary structure of NlCDK was predicted by SWISS-MODEL Workspace (Supplementary Figure S4). The binding mode of schaftoside and NlCDK1 protein was predicted by molecular docking analysis using DockingServer. The docking result suggested that schaftoside strongly interacts with NlCDK1 protein, promoted by -4.61 kcal/mol (ca. -19.36 kJ/mol) free energy of binding. Hydrogen bonds, polar groups and hydrophobic interactions are main forces contributing to schaftoside binding with NlCDK1. Schaftoside binds to the region of ATP binding element GXGXXGXV (Gly11–Val18) of NlCDK1 protein, at least through interacting with 3 amino acid residues Glu12, Thr14, and Val17 (**Figure 9** and **Table 2**). Schaftoside competed for binding to the phosphate site Thr14 by forming a strong hydrogen bond (O1-OG1), which indicated that the activation of NlCDK1 as a protein kinase should be seriously affected (**Table 2**). In addition, schaftoside also interacted with Lys34 and Arg36, blocking the cleft where the catalytic Lys33 located, and might affect the activity of NlCDK1 protein kinase (**Table 2** and Supplementary Figure S5).

**Table 2:** Interaction table of schaftoside and NlCDK1 protein.

Hydrogen bonds	Polar	Hydrophobic	Other
O1 (*1*)	O6 (*6*)	C15 (*29*)	C15 (*29*)
[*3.12*] – THR14 (*OG1*)	[*3.71*] – GLU12 (*OE2*)	[*3.61*] – VAL17 (*CG2*)	[*3.26*] – GLU12 (*CB*)
O12 (*12*)	H4 (*44*)	C12 (*26*)	O10 (*10*)
[*3.07*] – THR14 (*OG1*)	[*2.96*] – GLU12 (*OE2*)	[*3.77*] – VAL17 (*CG2*)	[*3.86*] – GLU12 (*CB*)
H8 (*48*)		C16 (*30*)	C10 (*24*)
[*3.40*] – THR14 (*OG1*)		[*3.61*] – VAL165 (*CB*)	[*3.68*] – GLU12 (*OE2*)
			H7 (*47*)
			[*2.78*] – THR14 (*CB, CG2*)
			C1 (*15*)
			[*3.75*] – THR14 (*OG1*)
			C5 (*19*)
			[*3.73*] – THR14 (*OG1*)
			C13 (*27*)
			[*3.76*] – THR14 (*CG2*)
			O11 (*11*)
			[*3.17*] – THR14 (*CG2*)
			O10 (*10*)
			[*3.59*] – VAL17 (*CG2*)
			C24 (*38*)
			[*3.85*] – LYS34 (*CE*)
			O14 (*14*)
			[*3.31*] – LYS34 (*CE*)
			C22 (*36*)
			[*3.57*] – ARG36 (*CB*)
			C24 (*38*)
			[*3.62*] – ARG36 (*CB*)

*Italic term in parentheses behind a elemental symbol and a number means frequency of the best geometry.*

*Italic terms in brackets is an RMSD value expressed in length units (Å).*

*Italic terms in parentheses behind a amino acid residue mean the group participated in forming the bond.*

### Effects of Schaftoside on the Activation of NlCDK1 Kinase

**FIGURE 10:**
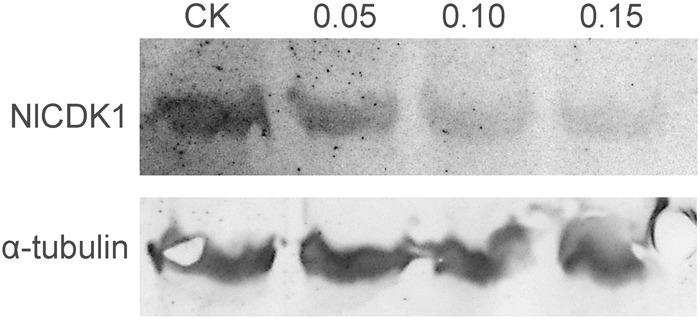
Western blot detects the phosphorylation at Thr-14 of NlCDK1 kinase.

The phosphorylation level on Thr-14 of NlCDK1 was probed with anti-phospho-CDK1 (pThr14) antibody, and the result showed that phosphorylation levels on Thr-14 of NlCDK1 generally decreased with the increase of schaftoside concentrations (**Figure 10**). When treated with 0.15 mg/ml schaftoside, phosphorylation on Thr-14 of NlCDK1 was almost decreased to an undetectable level. Phosphorylation on Thr-14 is one of the steps for the activation of CDK1 kinase, so the decrease of the phosphorylation level on Thr-14 suggested that the activation of NlCDK1 as a protein kinase was suppressed with schaftoside treatment.

## Discussion

Currently, characterization and identification of rice resistant genes against BPH have made great progress (Tamura et al., 2014). In contrast, identification of active compounds in rice against BPH are relatively weak, especially, little attention has been paid to the interaction of the active compound and its target in BPH. In this study, we identified a BPH resistance-associated compound schaftoside in rice, and found that schaftoside played its role in a dose dependent manner. The fluorescent spectra test showed that schaftoside has a strong ability to bind with NlCDK1. Docking model and western blot analysis confirmed that schaftoside treatment suppressed the phosphorylation on Thr-14 of NlCDK1, and as a result, inhibited the activation of NlCDK1 kinase. Most notably, this is the first study to our knowledge to investigate the interaction between schaftoside and NlCDK1. This study therefore unraveled a novel mechanism of rice resistance against BPH, and provided a valuable message for developing new strategy to control BPH.

In this study, total flavonoids were extracted and compared between different rice varieties. The results showed that rice plants including the susceptible varieties are rich in flavonoids. Further investigation found that the content of schaftoside is higher in resistant rice varieties than that in the susceptible, which indicates that schaftoside was implicated in rice resistance to BPH. Feeding experiments showed that schaftoside played its role in a dose dependant manner, which is similar to the previous findings using different rice lines (Grayer et al., 1994; Stevenson et al., 1996). We therefore suggested that the resistance of rice is closely related to the content of schaftoside, that is, rice varieties with low content of schaftoside are susceptible to BPH, while those with high content of schaftoside are resistant.

Fluorescent spectral experiment revealed that the concentration of schaftoside is an important element affecting the state of NlCDK1. The fluorescent intensity of NlCDK1 decreased with the increasing of schaftoside added, suggesting that more and more NlCDK1 was bound owing to the concentration of schaftoside went higher. To understand the molecular mechanism of schaftoside inaction with NlCDK1 protein, we performed molecular docking analysis, and the result suggested that schaftoside binds to the region of ATP binding element GXGXXGXV (Gly11 to Val18) of NlCDK1 protein, at least through interacting with 3 amino acid residues Glu12, Thr14, and Val17 (**Figure 9** and **Table 2**). CDK1 activity is regulated by phosphorylation and/or dephosphorylation on Thr14, Tyr15, and Thr161 (Parker and Piwnica-Worms, 1992). The ATP binding element GXGXXGXV of NlCDK1 contains 2 phosphate sites Thr14 and Tyr15 (**Figure 9** and Supplementary Figure S5), so the binding of schaftoside might affect the interacting of ATP and the regulating proteins upstream, such as wee1 and cdc25. Similarly, some flavonoids, such as chalcones and flavones, have also been reported as potent ATP competitive inhibitor of CDK1 (Navarro-Retamal and Caballero, 2016). Under a situation that schaftoside competed for binding to the phosphate site Thr14 by forming a strong hydrogen bond (O_1_-OG_1_), the activation of NlCDK1 as a protein kinase should be affected (**Table 2**). More importantly, western blot test using anti-phospho-CDK1 (pThr14) showed that phosphorylation levels on Thr-14 of NlCDK1 generally decreased with the increase of schaftoside concentrations, suggesting that schaftoside suppressed the activation of NlCDK1 kinase in a dose dependent manner (**Figure 10**). As described above, the resistant rice varieties contain more schaftoside, which indicates that BPH on resistant rice should gain more schaftoside from the plant. Therefore, with the accumulation of schaftoside ingested in BPH on resistant rice, more and more NlCDK1 would be bound and inhibited, and finally resulted in the death of BPH.

As described above, schaftoside affected the survival rate of BPH in a dose dependent manner. Molecular docking and western blot analysis strongly suggested that schaftoside suppressed the activation of NlCDK1 kinase, at least, by inhibiting the phosphorylation on Thr-14 site. Therefore, we think that higher concatenation of schaftoside would suppress more NlCDK1, and resulted in lower survival rate of BPH. Recently, we knockdown the expression of NlCDK1 using RNA interference technique, and found that NlCDK1 was required for the survival of BPH (Hao et al., 2018). However, it is still unclear how the inhibition of NlCDK1 resulted in the death of BPH. As is known, CDK1 is involved in diverse physiological processes, except for its famous role in cell cycle regulation. For example, CDK1 is also related to mitochondrial functions, including the adaptation to stress (Candas et al., 2013), regulation of mitochondrial preprotein translocase (Harbauer et al., 2014). It was also reported that CDK1 functioned in mitochondrial ATP generation, and dysfunction of CDK1 resulted in cell death (Rosenthal and Rosenthal, 2014; Li et al., 2016). Hence, a possibility is that CDK1 inhibition caused dysfunction of mitochondrial and decreases of ATP generation, and finally affected the survival of BPH. In the future, further study on how NlCDK1 inhibition affects the survival of BPH is needed, and some approaches like transcriptome or proteome analysis should be helpful in exploring the related genes, proteins, or pathways in responding to schaftoside treatment.

## Conclusion

In summary, we established HPLC fingerprints of the total flavonoids extracted from different rice varieties, and identified a resistance related compound as schaftoside using HPLC, MS/MS and NMR techniques. Schaftoside showed significant lethal effect on BPH in a dose dependent manner. The fluorescent spectra test and docking model suggested that schaftoside has a strong ability to bind with NlCDK1. Western blot analysis confirmed that schaftoside treatment suppressed the phosphorylation on Thr-14 of NlCDK1 kinase, and inhibited the activation of the kinase. Therefore, this research not only clarified a new mechanism of rice resistance against BPH by revealing the schaftoside-NlCDK1 interaction mode, but also provided a valuable message for developing some strategies to control BPH. In the future, it is a potentially important strategy to develop BPH-resistant rice with higher content of schaftoside in sustainable agriculture, through breeding hybrid varieties or constructing gene modified rice. It is also possible to design new insecticides with strategies like computer-aided drug design, based on the knowledge of interaction between schaftoside and its target protein NlCDK1.

## Author Contributions

P-YH and X-PY designed the research. P-YH, Y-LF, Y-SZ, YM, C-LY, X-MS, and H-LL performed the research or analyzed data. P-YH, Y-LF, Y-SZ, and X-PY prepared and edited the manuscript.

## Conflict of Interest Statement

The authors declare that the research was conducted in the absence of any commercial or financial relationships that could be construed as a potential conflict of interest.

## Footnotes

1 http://www.ncbi.nlm.nih.gov/

2 https://www.swissmodel.expasy.org

3 https://www.dockingserver.com/web

4 http://www.wwpdb.org

## Abbreviations

BPH: brown planthopper
HPLC: high performance liquid chromatography
MS: mass spectrometry
NMR: nuclear magnetic resonance
