# Supplementary material for: Schaftoside Interacts With NlCDK1 Protein: A Mechanism of Rice Resistance to Brown Planthopper, Nilaparvata lugens
Source: Front Plant Sci. 2018 May 29;9:710. doi: 10.3389/fpls.2018.00710 (PMC5986872; doi:10.3389/fpls.2018.00710)

Figure S2. MS and MS/MS for Peak 3

MS

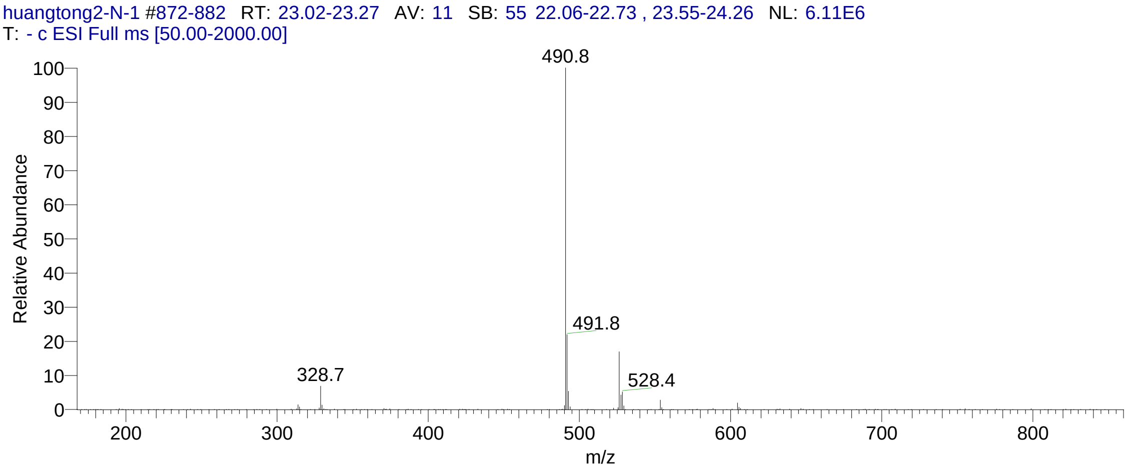

MS/MS

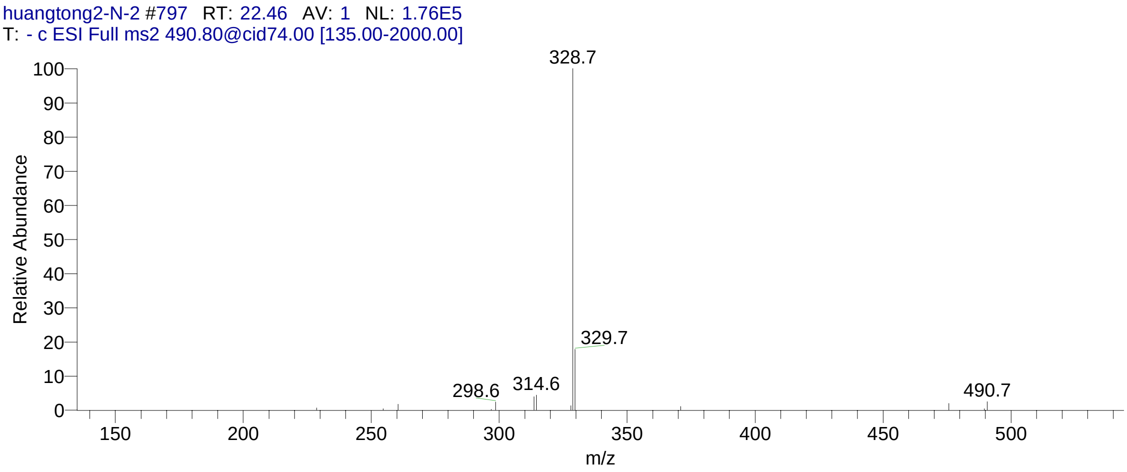

Supplement: Supplementary file 2 [file Image_2.PDF]
