# Supplementary material for: Schaftoside Interacts With NlCDK1 Protein: A Mechanism of Rice Resistance to Brown Planthopper, Nilaparvata lugens
Source: Front Plant Sci. 2018 May 29;9:710. doi: 10.3389/fpls.2018.00710 (PMC5986872; doi:10.3389/fpls.2018.00710)

Figure S3. MS and MS/MS for Peak 4

MS

huangtong2-N-1 #1163-1173 RT: 30.72-30.98 AV: 11 SB: 78 29.35-30.45 , 31.45-32.39 NL: 3.42E6
T: - c ESI Full ms [50.00-2000.00]

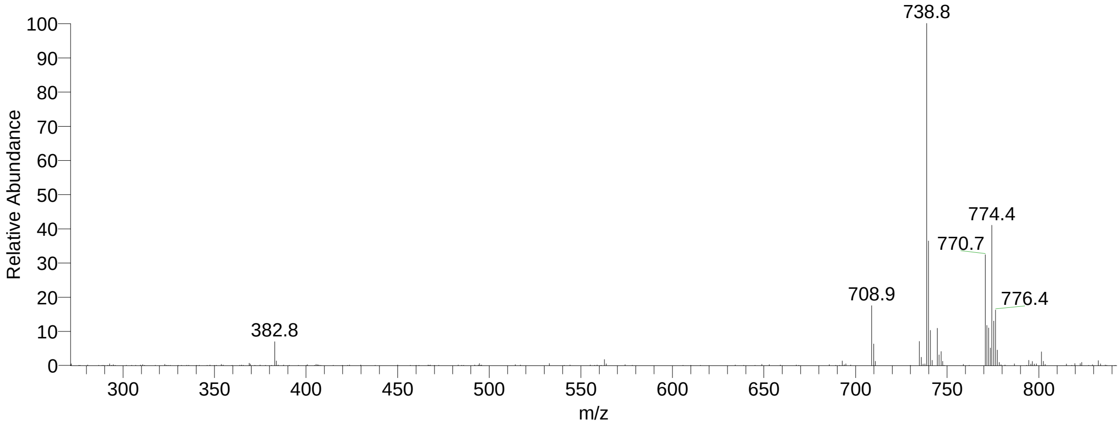

MS/MS

huangtong2-N-2 #983-985 RT: 30.50-30.57 AV: 3 NL: 5.20E5
T: - c ESI Full ms2 738.80@cid74.00 [200.00-2000.00]

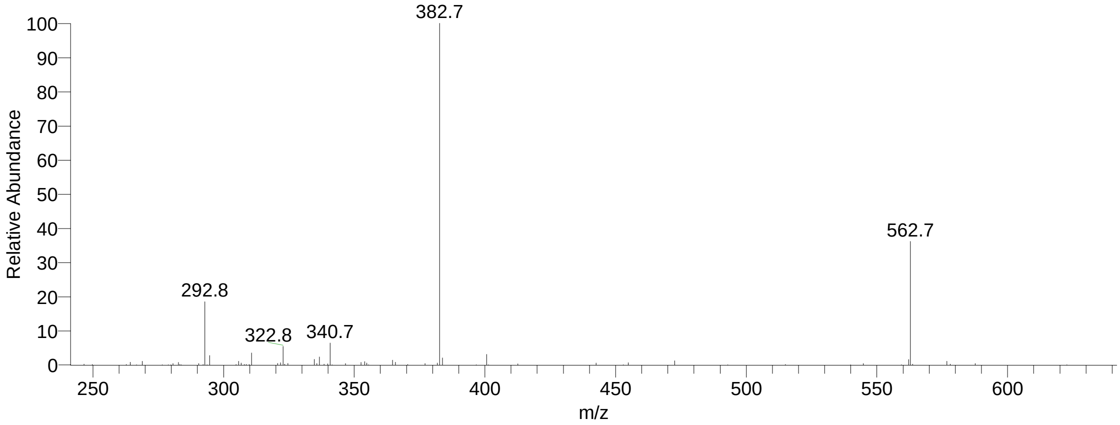

Supplement: Supplementary file 3 [file Image_3.PDF]
