# Supplementary material for: Schaftoside Interacts With NlCDK1 Protein: A Mechanism of Rice Resistance to Brown Planthopper, Nilaparvata lugens
Source: Front Plant Sci. 2018 May 29;9:710. doi: 10.3389/fpls.2018.00710 (PMC5986872; doi:10.3389/fpls.2018.00710)
Supplement: Supplementary file 4 [file Image_4.PDF]

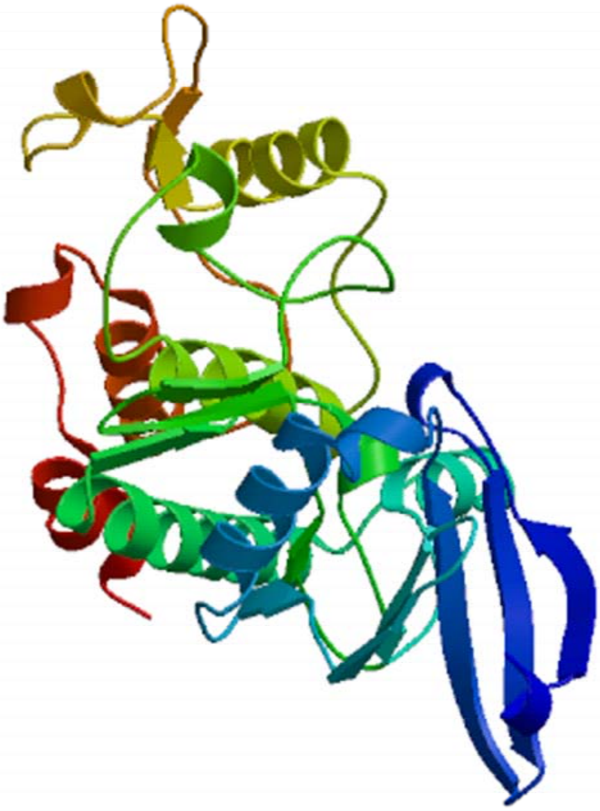

Supplementary Figure S4. Tertiary structure of NlCDK. The tertiary structure was predicted by SWISS-MODEL Workspace, based on the available structure of CDK1 protein (PDB entry code: 4y72.1.A).
