# Supplementary material for: Schaftoside Interacts With NlCDK1 Protein: A Mechanism of Rice Resistance to Brown Planthopper, Nilaparvata lugens
Source: Front Plant Sci. 2018 May 29;9:710. doi: 10.3389/fpls.2018.00710 (PMC5986872; doi:10.3389/fpls.2018.00710)
Supplement: Supplementary file 5 [file Image_5.PDF]

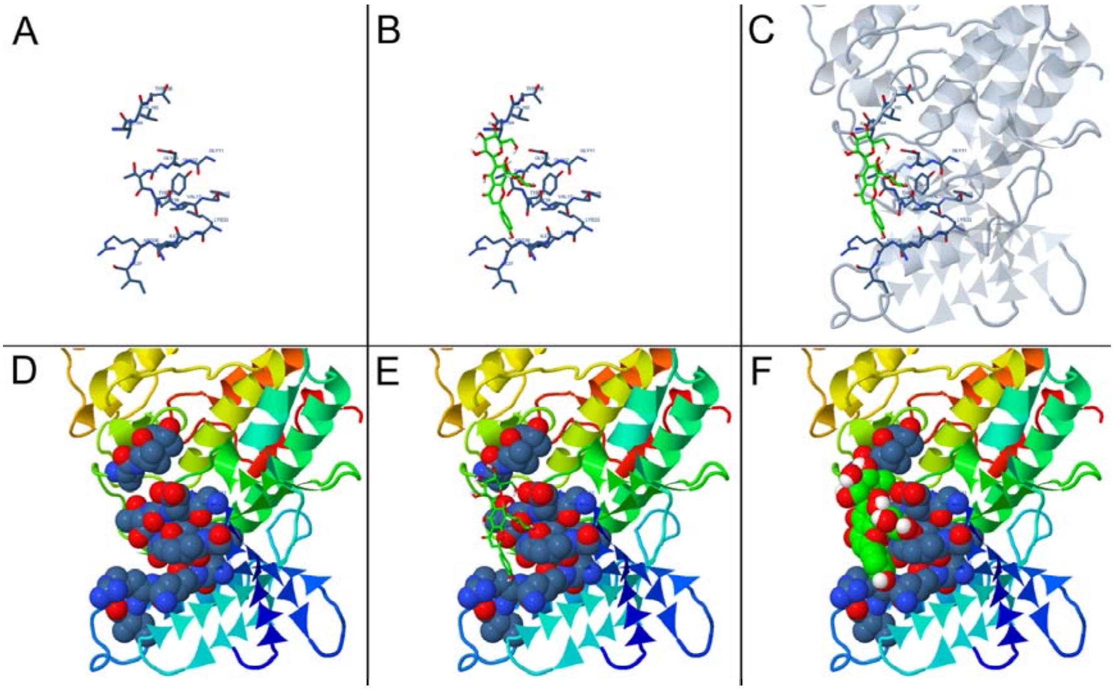

Supplementary Figure S5. Binding mode of schaftoside to NlCDK1. (A), amino acid residues near the binding regions of NlCDK1, without binding schaftoside. (B), (C), (E) and (F), schaftoside binding with NlCDK1. In (C), gray color represented for non-binding regions of NlCDK1 (local spacial structure). In (D), (E) and (F), non-binding regions of NlCDK1 are shown in color to substitute for gray in (C). Carbon atoms in NlCDK1 and schaftoside are shown in blue and green, respectively. Colors of other atoms are as follows: oxygen (red), nitrogen (light blue).
